# User perspective and higher cognitive task-loads influence movement and performance in immersive training environments

**DOI:** 10.1186/s42490-019-0021-0

**Published:** 2019-08-30

**Authors:** Juan Trelles Trabucco, Andrea Rottigni, Marco Cavallo, Daniel Bailey, James Patton, G. Elisabeta Marai

**Affiliations:** 1 Department of Computer Science, University of Illinois at Chicago, 851 S Morgan Street, Chicago, 60607 USA; 2 Department of Bioengineering, University of Illinois at Chicago, 851 S Morgan Street, Chicago, 60607 USA

**Keywords:** User perspective, Engagement, CAVE2

## Abstract

**Background:**

In virtual reality (VR) applications such as games, virtual training, and interactive neurorehabilitation, one can employ either the first-person user perspective or the third-person perspective to perceive the virtual environment; however, applications rarely offer both perspectives for the same task. We used a targeted-reaching task in a large-scale virtual reality environment (*N*=30 healthy volunteers) to evaluate the effects of user perspective on the head and upper extremity movements, and on user performance. We further evaluated how different cognitive challenges would modulate these effects. Finally, we obtained the user-reported engagement level under the different perspectives.

**Results:**

We found that first-person perspective resulted in larger head movements (3.52±1.3*m*) than the third-person perspective (2.41±0.7*m*). First-person perspective also resulted in more upper-extremity movement (30.08±7.28*m* compared to 26.66±4.86*m*) and longer completion times (61.3±16.4*s* compared to 53±10.4*s*) for more challenging tasks such as the “flipped mode”, in which moving one arm causes the opposite virtual arm to move. We observed no significant effect of user perspective alone on the success rate. Subjects reported experiencing roughly the same level of engagement in both first-person and third-person perspectives (*F*(1.58)=0.9,*P*=.445).

**Conclusion:**

User perspective and its interaction with higher-cognitive load tasks influences the extent of movement and user performance in a virtual theater environment, and may influence the choice of the interface type (first or third person) in immersive training depending on the user conditions and exercise requirements.

## Background

Interactive virtual reality (VR) applications that engage human motor performance span a wide variety of domains, including piloting remote vehicles, movement performance training, and neurorehabilitation therapy following brain injury. These applications often seek to stimulate brain activity, facilitate practice-based learning, and to leverage the brain’s capability for reorganizing its structure [1]. In this sense, using a display to provide visual feedback to the trainer or trainee can reinforce learning and performance skills. As technology evolves, new opportunities for trainee feedback become possible. For example, out-of-body perspectives in immersive environments allow for motion feedback that may otherwise not be visible or apparent to the trainee. However, whether such interactive approaches impact the user performance and mental load is an open research question. For instance, it is not currently known which characteristics of virtual reality are most beneficial for rehabilitation training [2], although one avenue might be to increase the patient’s engagement with the therapy [3]. Engagement, as defined in the videogame literature, is the experience involving a lack of awareness of time and the real world, and a sense of being in the task environment [4].

From the multiple VR characteristics to consider, the user perspective is of particular interest, since it can dramatically influence the user’s perception of their body within the environment. A first-person perspective presents the world directly through the character’s eyes, though sometimes without a body representation. In contrast, a third-person perspective, while not natural, allows a full view of body motions. In our study, we displayed the virtual character in front of the user’s viewpoint; the avatar did not act as a mirror. Three studies have compared these user perspectives in general VR applications. Salamin’s experiments using a head-mounted display endorsed the use of the first-person perspective for precision tasks and the use of third-person perspective for movement tasks [5]. Later, Salamin found that participants attempting to catch a ball estimated distances better and performed more similarly to real world-conditions in third-person perspective [6]. Covaci et al. [7] compared the perspectives in a cave automatic virtual environment (CAVE) with a ball throwing task. In this task, participants underestimated distances in both perspectives; however, similarly to Salamin results, they performed slightly better in third-person perspective. It remains unclear what other effects if any, the viewer perspective may have on aspects like motion or perceived engagement and whether VR applications in some domains justify their choice (e.g., limb-studies [8]).

Not only the engagement can vary depending on the user perspective but also based on the chosen immersive environment. Immersive environments use stereoscopic imagery to enhance depth perception and position tracking to react to user’s movement, being the most common head-mounted displays and small rooms with projectors [9]. Another environment is a virtual-reality theater where instead of using projectors, the setup uses tiled-displays. Compared to head-mounted displays, a virtual-reality theater provides a nonintrusive and collaborative experience (although not in this study), only requiring the users to wear lightweight polarized glasses. Besides, virtual-reality theaters provide higher-resolution than most of the commercial head-mounted displays and allow the user to see the real world. Despite the potential benefits of these virtual-reality theaters to a variety of interactive training, few studies have yet explored such benefits.

In this work, we evaluate the relationships between user perspective and user performance, motion, and perceived level of engagement. From a motor rehabilitation perspective, such an evaluation would be enriched by further considering the context of cognitive load levels. Therefore, we test under varying cognitive loads the existing null hypothesis that user perspective does not influence user performance, body head, and upper-extremity movements, or self-perceived level of engagement. To test this hypothesis, we use a virtual target-reaching game to evaluate the effects of both first- and third-person perspectives in a virtual reality theater environment, a CAVE2 [10]. The game makes possible the use of different challenge levels, leading to exercises with varying cognitive loads.

## Results

### Head movements

**Fig. 1:**
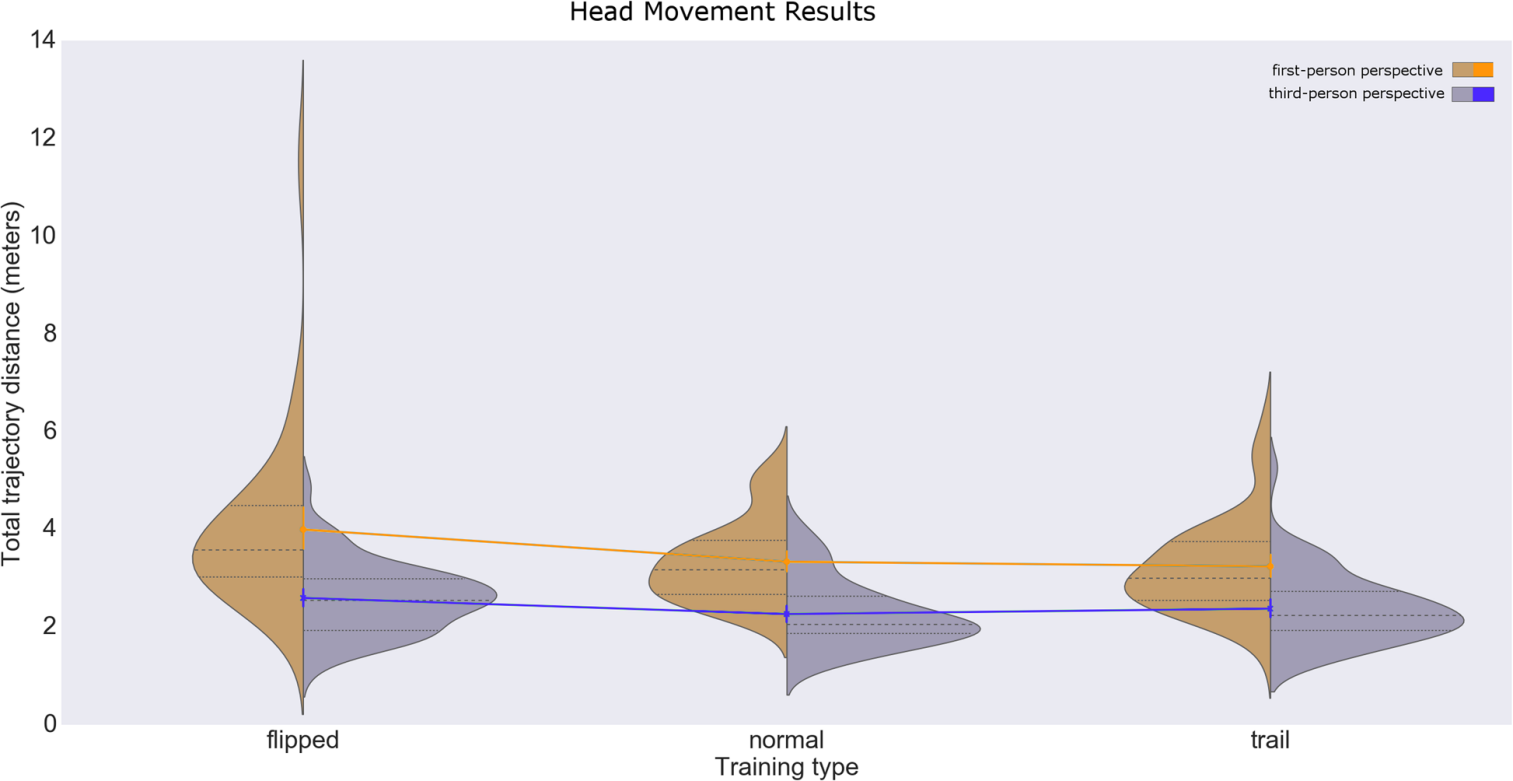
Head movement results. Distribution plots for the total head trajectory distance after 20 attempts, for each exercise type and user perspective. The two colored polylines show the mean of each perspective distribution, and its variation with the exercise type

We found that first person user perspective caused larger head movements (3.52±1.3*SD* m) than third-person perspective (2.41±0.7*SD* m; main effect *F*(1,29)=159.2, *P*<.0001; see Fig. 1). The flipped exercise head movement was also significantly higher than both normal and trail exercises in first-person (*F*(2,58)=19.4,*P*<.0001) and similarly in third-person. Data distribution passed the normality test (*P*=0.064) and sphericity assumption after the application a Box-Cox transformation. Results statistics are available in Table 1.

**Table 1:** Results for head movements (in meters)

User perspective	Exercise type	Target size	Mean	STD
First person	Normal	10 cm	3.40	0.78
First person	Flipped	10 cm	4.19	1.83
First person	Trail	10 cm	3.26	0.90
Third person	Normal	10 cm	2.28	0.68
Third person	Flipped	10 cm	2.59	0.73
Third person	Trail	10 cm	2.32	0.59
First person	Normal	15 cm	3.28	0.87
First person	Flipped	15 cm	3.84	1.66
First person	Trail	15 cm	3.22	1.05
Third person	Normal	15 cm	2.25	0.69
Third person	Flipped	15 cm	3.18	1.18
Third person	Trail	15 cm	2.43	0.83
First person	Normal	All	3.34	0.83
First person	Flipped	All	4.00	1.78
First person	Trail	All	3.24	0.97
Third person	Normal	All	2.27	0.68
Third person	Flipped	All	2.60	0.70
Third person	Trail	All	2.38	0.71
First person	All	All	3.52	1.30
Third person	All	All	2.41	0.70

### Upper-extremity movements

**Fig. 2:**
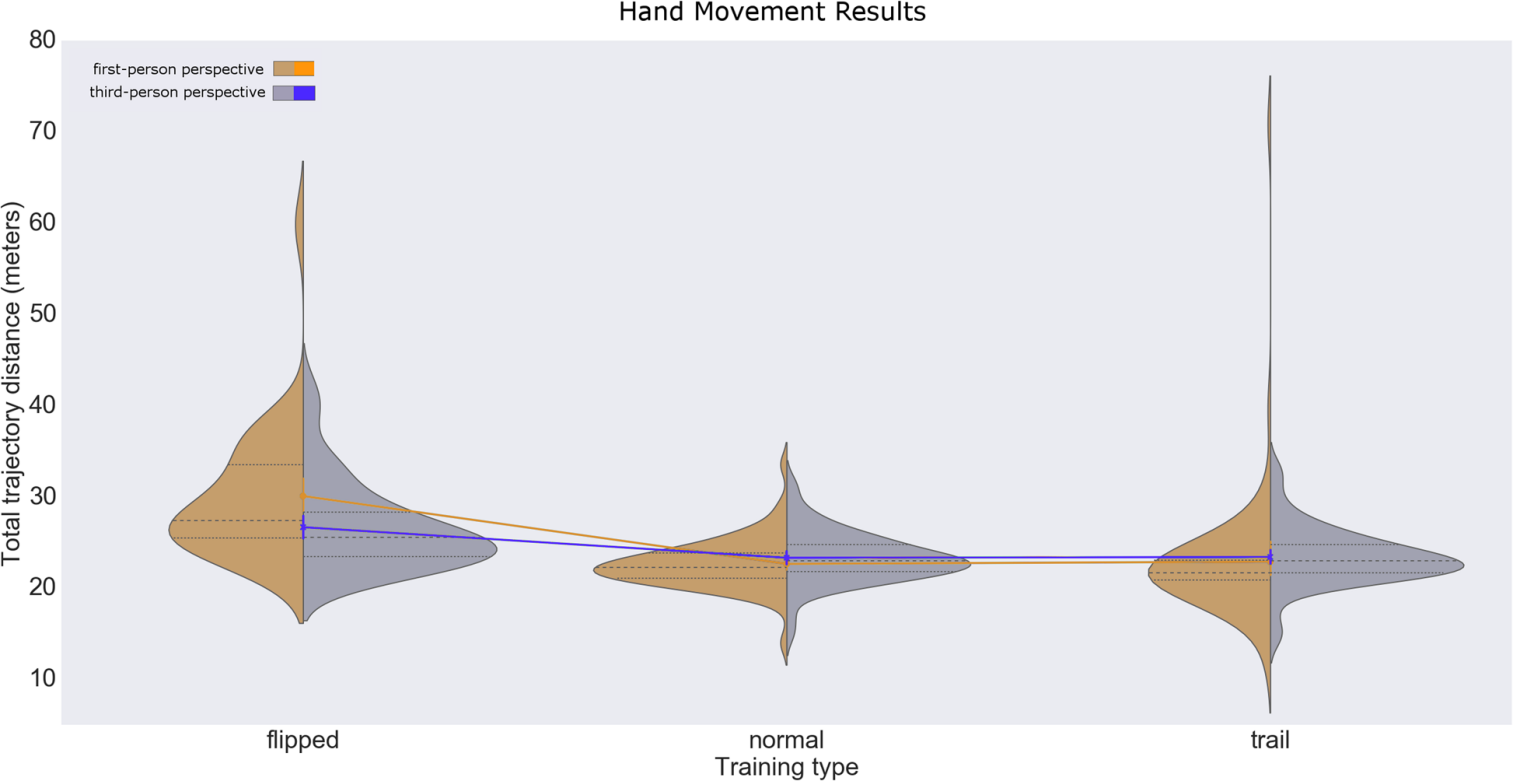
Hand movement results. Distribution plots for the total trajectory distance after 20 attempts, for each exercise type and user perspective

Exercise type caused the largest effect on the upper-extremity movement (F(2319) = 168.4, *P*<.0001). The flipped exercise mode lead to longer trajectories: flipped exercises (Fig. 2) in first-person perspective (30.08±7.28*SD* m) had longer trajectories than flipped exercises in third-person perspective (26.66±4.86*SD* m). The second effect was caused by the interaction between the perspective and exercise type (*F*(2319)=25.6,*P*<.0001).

We noted that the displayed trail paths did not influence the movement chosen by a subject; none of the subjects followed a straight line, but rather the expected geodesic curve. We further noticed that in the flipped exercise mode, different subjects adopted different strategies when negotiating the higher cognitive load: some subjects aimed for the targets directly, while others explored the space from the frontal plane towards the sagittal plane, to first scan the area. Results statistics are available in Table 2.

**Table 2:** Results for upper-extremity movements (in meters)

User perspective	Exercise type	Target size	Mean	STD
First person	Normal	10 cm	22.51	3.08
First person	Flipped	10 cm	30.20	7.16
First person	Trail	10 cm	22.33	4.33
Third person	Normal	10 cm	23.01	2.97
Third person	Flipped	10 cm	26.58	5.09
Third person	Trail	10 cm	23.27	3.13
First person	Normal	15 cm	22.77	2.42
First person	Flipped	15 cm	29.96	7.54
First person	Trail	15 cm	23.43	9.40
Third person	Normal	15 cm	23.61	2.59
Third person	Flipped	15 cm	26.74	4.70
Third person	Trail	15 cm	23.53	3.20
First person	Normal	All	22.64	2.75
First person	Flipped	All	30.08	7.28
First person	Trail	All	22.88	7.28
Third person	Normal	All	23.31	2.78
Third person	Flipped	All	26.66	4.86
Third person	Trail	All	23.40	3.14
First person	All	All	25.20	7.03
Third person	All	All	24.46	4.01

### Completion time

**Fig. 3:**
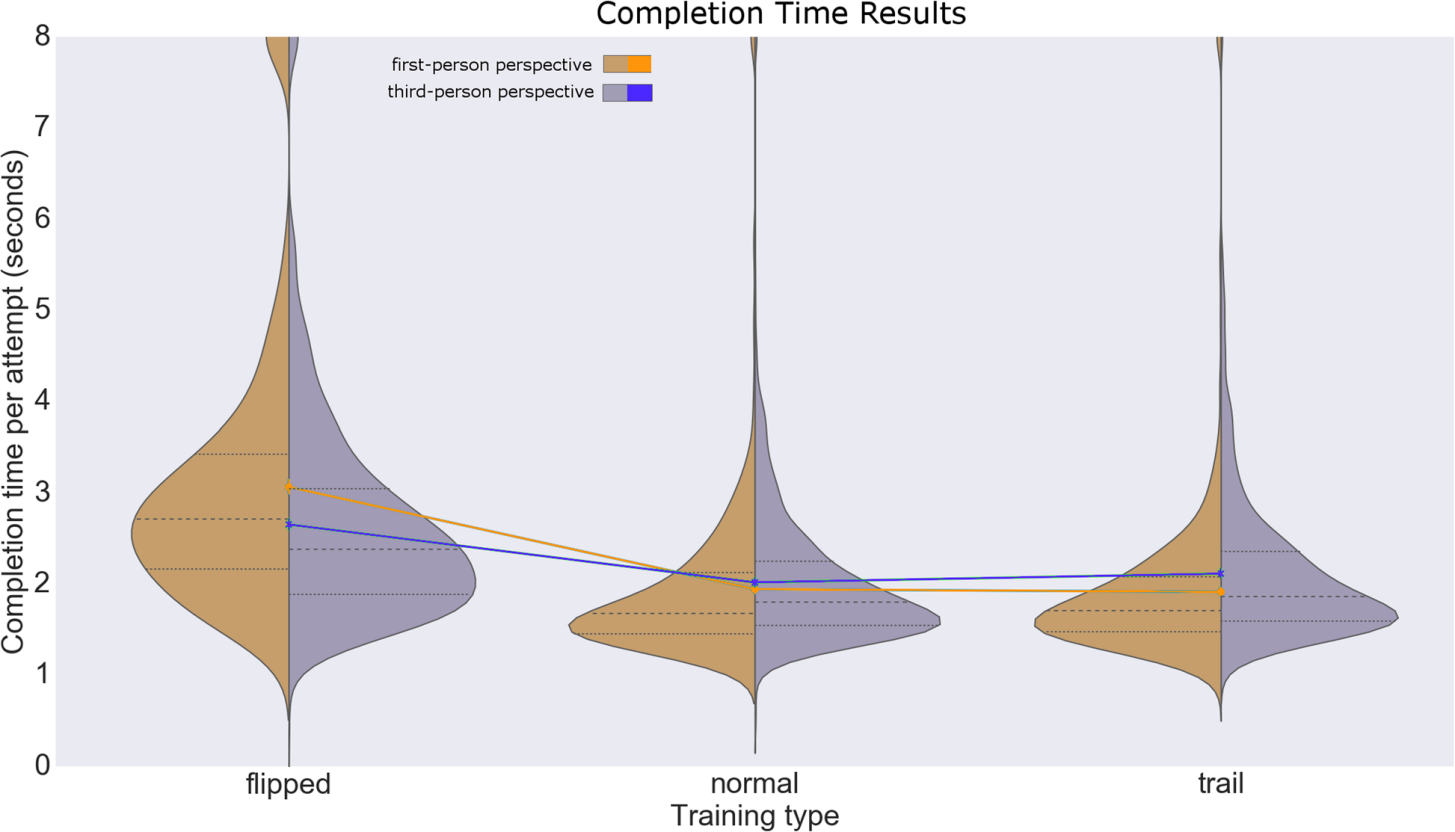
Completion time results. Completion-time distribution plots for each exercise type and user perspective

The results show complex variation in the completion time, related to both user perspective (first or third) and exercise type (normal, trail, or flipped) (Fig. 3). Surprisingly, perspective effects varied interactively with the exercise type (ANOVA main effect *F*(2,58)=39.7,*P*<.0001). In particular, for the normal exercise type, the third person completion times were the same as the first person times. For the trail type, the third person times were higher than the first person times (M_trail_third=42.2s, SD_trail=6.8s; M_trail_first = 38.2s, SD_trail_first=11.2s). For the flipped type, the third person times were lower than the first person times (M_flipped_third=53s, SD_flipped_third=10.4s; M_flipped_first=61.3s; SD_flipped_first=16.4s).

The largest effect on completion time was caused by the flipped exercise type, which lead to the largest completion time (*F*(2,58)=200.4,*P*<.0001, followed by pairwise post hoc comparisons; Fig. 3). User perspective also influenced completion time (*F*(1,29)=7.5,*P*=.01, followed by pairwise post hoc comparisons; Fig. 3). Target size apparently also influenced completion time (*F*(1,29)=10.9,*P*=.003); although the ANOVA results suggested an effect of the target sphere size, we did not find significant differences in the post hoc analysis. We further found interactions between perspective and target size (*F*(1,29)=6.8,*P*=.015). The data passed the Lilliefors test (*P*=.094) after performing a Box-Cox transformation, and did not violate the sphericity assumption. Results statistics are available in Table 3.

**Table 3:** Results for completion time (in seconds)

User perspective	Exercise type	Target size	Mean	STD
First person	Normal	10 cm	40.7	11.6
First person	Flipped	10 cm	63.7	16.6
First person	Trail	10 cm	38.6	7.7
Third person	Normal	10 cm	40.3	5.2
Third person	Flipped	10 cm	53.8	10.3
Third person	Trail	10 cm	42.1	6.3
First person	Normal	15 cm	37.0	5.3
First person	Flipped	15 cm	58.8	16.1
First person	Trail	15 cm	37.9	14.0
Third person	Normal	15 cm	40.3	5.8
Third person	Flipped	15 cm	52.3	10.5
Third person	Trail	15 cm	42.4	7.4
First person	Normal	All	38.8	9.1
First person	Flipped	All	61.3	16.4
First person	Trail	All	38.2	11.2
Third person	Normal	All	40.3	5.4
Third person	Flipped	All	53.0	10.4
Third person	Trail	All	42.2	6.8
First person	All	All	46.1	16.5
Third person	All	All	45.2	9.6

### Hitting the target - score

**Fig. 4:**
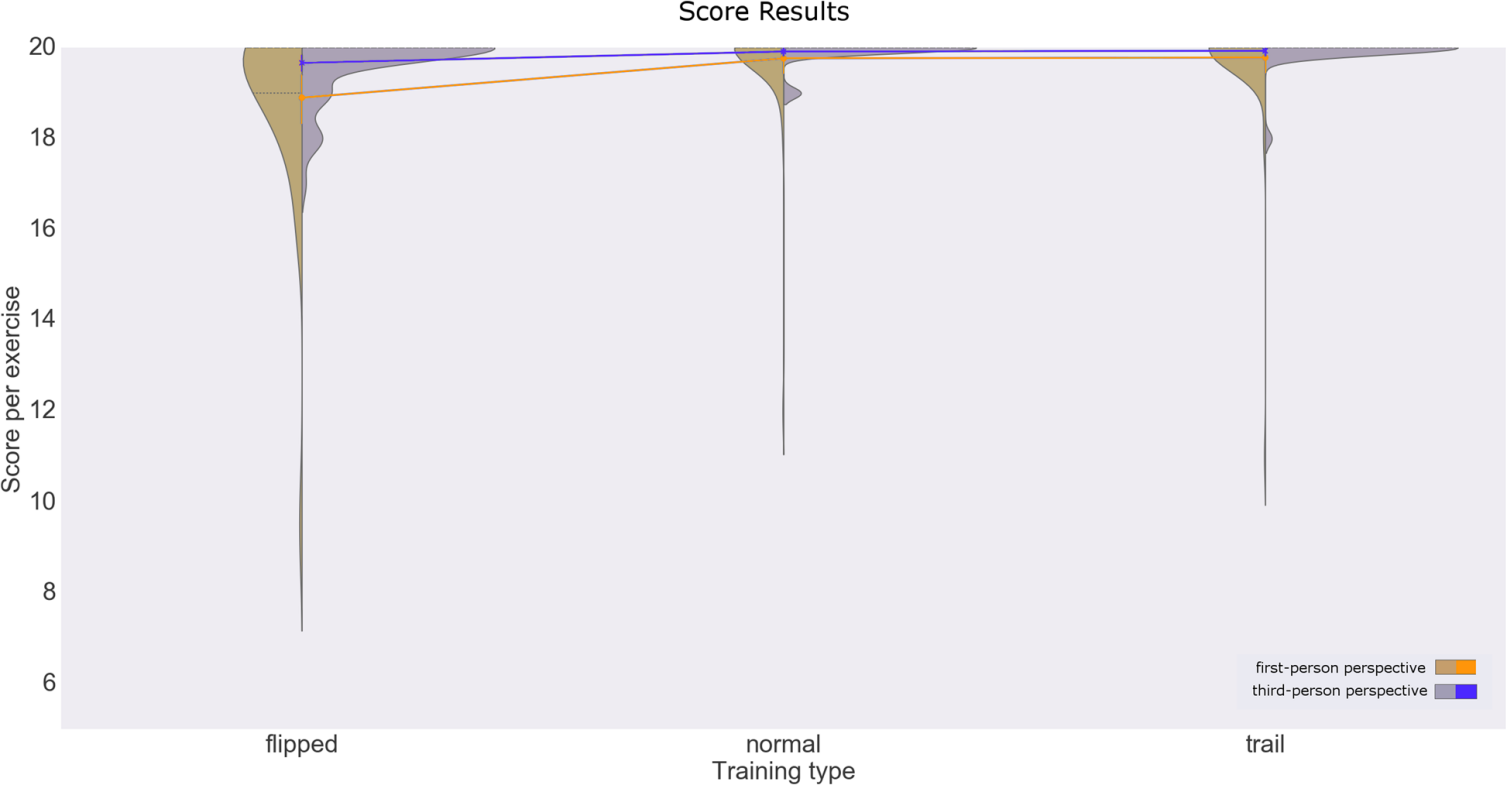
Score results. Score distribution plots for each exercise type and user perspective

Subjects obtained high scores consistently in all three exercise types (*M*=19.66,*MED*=20,*MIN*=9,*MAX*=20), in both first and third-person perspectives. Again, we found differences due to multiple factors. User perspective caused the largest effect on the score (*F*(1319)=86.4, *P*<.0001); the first-person perspective correlated with lower scores in flipped mode (*M*=18.9,*SD*=2.1) in comparison to the third-person perspective (*M*=19.9, *SD*=0.7). The exercise type caused the second largest effect on the score (*F*(2319)=37.1,*P*<.0001); scores for exercises in flipped mode were significantly lower than in the normal and trail modes. Results also suggest the effect of other factors on the score, such as the target size (*F*(1319)=30.7,*P*<.0001), the interaction between perspective and exercise type (*F*(2319)=31.2,*P*<.0001), the interaction between perspective and target size (*F*(1319)=14.5,*P*=.0002), and the interaction of all the factors (*F*(2319)=3.25,*P*=.04). However, the post hoc analysis indicated that flipped exercises were the most common factor associated with subjects missing targets (Fig. 4). Additionally, the skewed distribution to the right (higher scores) suggests the reaching exercise game had overall low difficulty. Results statistics are available in Table 4.

**Table 4:** Results for hitting the target - score

User perspective	Exercise type	Target size	Mean	STD
First person	Normal	10 cm	19.6	1.5
First person	Flipped	10 cm	18.7	2.3
First person	Trail	10 cm	19.9	0.5
Third person	Normal	10 cm	19.9	0.3
Third person	Flipped	10 cm	19.7	0.7
Third person	Trail	10 cm	20.0	0.0
First person	Normal	15 cm	19.9	0.4
First person	Flipped	15 cm	19.1	1.9
First person	Trail	15 cm	19.7	1.6
Third person	Normal	15 cm	19.9	0.3
Third person	Flipped	15 cm	19.7	0.6
Third person	Trail	15 cm	19.9	0.5
First person	Normal	All	19.8	1.1
First person	Flipped	All	18.9	2.1
First person	Trail	All	19.8	1.2
Third person	Normal	All	19.9	0.3
Third person	Flipped	All	19.7	0.7
Third person	Trail	All	19.9	0.4
First person	All	All	19.5	1.6
Third person	All	All	19.8	0.5

### Level of engagement

**Fig. 5:**
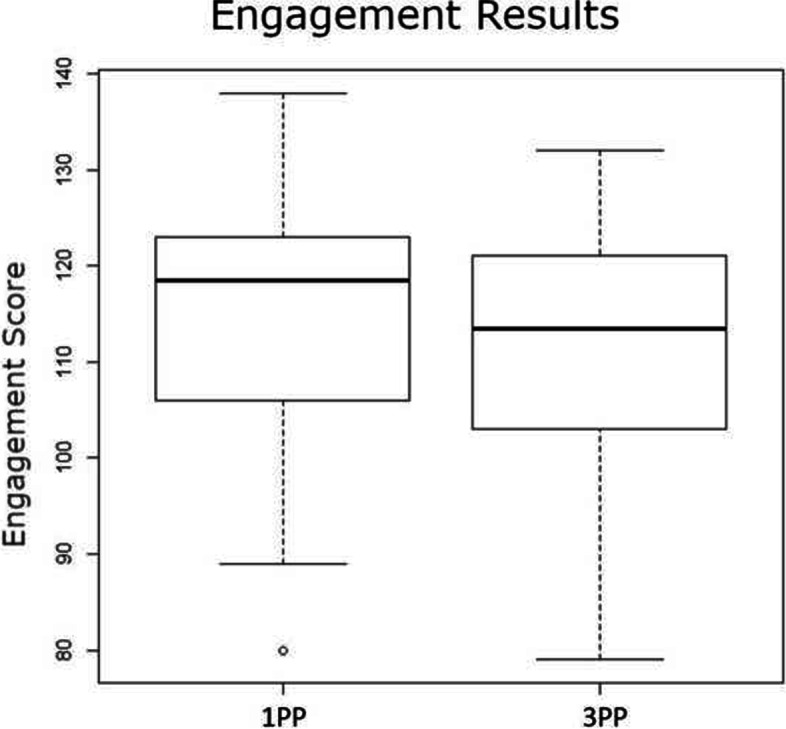
Engagement results Engagement score for first-person perspective and third-person perspective

We found no significant differences in the engagement questionnaire score between first and third-person perspectives (*F*(1,58)=0.59,*P*=.445; Fig. 5). Non-parametric analysis also revealed no significant differences (Kruskal-Wallis test, *X*^2^=2.72,*P*=.0989). The data distribution transformed with the Box-Cox function satisfied the normality test (*P*=.1192) and the Levene’s test (*P*=.766). Results statistics are available in Table 5.

**Table 5:** Results for level of engagement (from questionnaire)

User perspective	Mean	STD
First person	114.3	23.6
Third person	111.7	23.9

## Discussion

### Body movements

As shown by our results, we found differences in head and hand movements depending on the user perspective and the three exercise types. These differences are of particular importance for VR-based rehabilitation, where the patient’s condition constrains the range of motion that patient can perform.

The user perspective had the main effect on the head movements; there was significantly longer total distance traveled by the head over 20 reaching attempts in the first-person (*M*=3.52, SD = 1.30) perspective than third-person perspective (*M*=2.41, SD = 0.70). Compared to the third-person perspective, in which the avatar and targets appear in front of the user, the first-person perspective required the subjects to explore a wider area of screen space, which implicitly required the use of peripheral vision. From the perspective of VR-based rehabilitation, the use of the first-person perspective may be beneficial when the patient requires broader spatial exploration as in rehabilitation of spatial neglect [11].

For upper-extremity movements, the first-person perspective with higher cognitive load exercises (i.e., flipped) yielded longer trajectories. In contrast, upper-extremity movements for the normal and trail exercise settings did not reveal significant differences between the two user perspectives; thus, revealing a zero-effect from the visual cues shown as trails and the shortest path between the hand and target on the trajectory distance.

### User performance

**Fig. 6:**
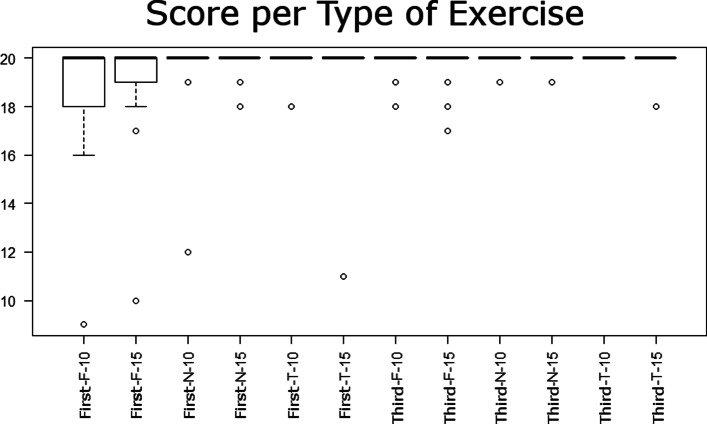
Boxplot of the score per exercise combination. Scores computed as number of targets touched for all the combination of exercises in 20 attempts: First-person perspective or third-person perspective executed in normal (N) mode, flipped (F) mode or trail (T) mode with targets of 10 or 15 cm of diameter

Our results indicate that the user perspective alone is not the main factor influencing user performance, but a combination of user perspective and cognitive load. As expected, we found that flipped exercises (higher cognitive load) required more time to be completed (Fig. 3) in both user perspectives and had lower success rates (Fig. 6), compared to the normal and trail exercises. However, for this higher cognitive load exercise, subjects took more time to complete the task and had lower task success rates in the first-person perspective than in the third-person perspective. For normal exercises, there were no differences between user perspectives. For trail exercises, subjects took longer to complete the task in third-person perspective compared to first-person perspective (opposite case than trail exercises) and had similar success rates between the two perspectives. Similar to Salamin [6], user perspective alone did not influence completion time, and similar to Covaci et al. [7], user perspective alone did not influence task success.

We believe that the opposite results in completion time between the higher cognitive load task and the trail task relies on two factors: movements feedback and environmental feedback. In third-person perspective, the subjects were able to instantly observe wrong actions in the avatar and make corrections accordingly while in the first-person perspective, the restricted field of view did not ease this immediate feedback. This movement feedback was particularly useful for the higher cognitive task, the most unfamiliar for the subjects. As for environmental feedback, in the trail exercise, the displayed segment connecting the closest hand to the target was more visible in the first-person perspective; thus, it caught the subject’s attention faster. These visual cues may also be helpful to get people’s attention back, especially for patients with spatial neglect. Opposed to Salamin’s preference for using the first-person perspective for precision tasks [5], we believe that the third-person perspective feedback is preferred for precision tasks with a higher cognitive load that require unfamiliar movements and performed on an environment with field of view constraints. The recommendation may not apply to other cognitive tasks such as memory attention unless mixed in a dual attention setup with unfamiliar movements.

### Level of engagement

In this study, we found that the user perspective had no significant effect on the degree of user-perceived engagement. Our findings are supported by the conflicting results reported by Denisova and Cairns [12] (first-person perspective more immersive) and respectively by Salamin [5] (third-person perspective more engaging). We believe that the low degree of task difficulty (for a healthy subject) and the static foot positioning required by the rehabilitation application may have influenced the subjects’ sense of engagement. As suggested by Faria et al. [13], exercises requiring more cognitive effort and attention may gather more attention. On the other hand, the study of Schuurink and Toet [14] suggests that the level of stimulation induced by a virtual reality environment is independent of the question of first- and third-person perspectives; our results support Schuurink and Toet’s conclusion in the context of tasks with relatively reduced cognitive effort.

We looked into the self-perceived level of engagement due to its potential to improve a patient’s experience with rehabilitation tasks. To measure this we used the definition of engagement from the gaming literature, while implementing our experiments in an immersive VR environment. We note that the term ‘immersion’ is overloaded between gaming research and VR research. Gaming research operates under the definition given by Jennet et al. [4], who describe the different levels of engagement as immersion, an experience felt by gamers. In the gaming literature, immersion is therefore used interchangeably with engagement and involvement [15]. Jennet et al. further created the IEQ questionnaire to assess immersion (defined as engagement), based on components that influence the gamer experience. In contrast, VR research uses the term immersion to describe a property of the technology [16] that influences the level of presence — the “illusion of being there, notwithstanding that you know for sure that you are not” [17]. Each of the two definitions of immersion is equally influential in its field of origin. Because we seek to measure game engagement, in our questionnaire we followed the Jennet et al. [4] definition. This definition acknowledges the influence of presence on engagement, and suggests that presence only appears at the deeper level of engagement.

### Depth perception errors

Based on the questionnaire feedback, in the third-person perspective a few subjects experienced misperceptions, with no further impact, for objects placed to the sides and close to the frontal plane. Two of the subjects reported that in the third-person view the displayed scene seemed to be rendered in 2D; another subject thought mistakenly that the objects were behind the avatar. It is possible the lack of geometrical visual cues in spherical targets contributed to these isolated depth perception issues, although we provided consistently shadows as a cue. For example, Powell et al. [18] suggest using complex geometries (more complex than our simple sphere geometry) to further provide visual cues for reaching tasks. Nevertheless, none of these reported ln appear to have resulted in lower user performance than in the first-person view.

Conversely, no subjects reported depth-perception errors in first-person perspective, despite lower user performance results under specific settings. A recent study [19] in CAVE-like environments suggested that targets near the screen—that hence appear large to the user (as in first-person perspective)—are easier to understand than objects far into the scene (as in third-person perspective). Results from the Bruder et al. study [19] further report up to 50% misinterpretations of distance for objects that are further away. We believe their findings help explain the difference in our user reports of depth-perception errors.

### Assumptions and limitations

Concerning assumptions and limitations, our study used a specific infrastructure that is currently not readily accessible to the public due to cost, technical support, and space requirements. We note, however, that the CAVE2 merely serves as a vehicle for testing our hypothesis, since it allows both first person and third person large screen feedback display, aside from the CAVE2’s many other, vast capabilities, which were not used in this study. We believe these findings would transfer to any another immersive system that allows both first person and third person large screen display. Given a strong need in domains like rehabilitation, such systems are not inaccessible to rehabilitation clinics. Our findings may also not be directly transferable to other platforms, for example, head-mounted displays. However, immersive large environments have specific advantages over head-mounted displays (e.g., non-intrusive equipment) that make such environments relevant to rehabilitation clinics. Last but not least, as suggested by Levin et al. [20], in general, further evaluation of the performance, quality and surrogate aspects of motor behavior are needed to analyze the fidelity of VR environment tasks to physical environment tasks. We note, nevertheless, on the deliberate simplicity of the exercise set we designed and we tested in this work, in collaboration with a domain expert.

For our study design, even though we randomized the starting user perspective per subject, the order of the exercises with different target sizes was not randomized which could introduce confounding effects. Moreover, our study considers engagement from the perspective of a self-reported “experience that produces a lack of awareness of time and the real world” [4]. We do not consider other aspects of the virtual reality experience such as embodiment (the sensation of being inside a body) [21].

The success score median of 20 (out of 20) across all exercises and both perspectives indicates that our game had an easy level of difficulty for healthy subjects. We corroborated this last statement with the results of the user survey; on a 1 to 5 Likert scale from easy to difficult, subjects scored the first-person perspective as 2.04 ± 0.92 and the third-person perspective as 2.21 ± 0.92. These results are not surprising as the arm-reaching exercises were designed for physical therapy patients, but tested on healthy subjects; rehabilitation patients would find the exercises more difficult. Furthermore, we allowed the healthy subjects eight seconds to target each object, when in fact they only needed an average of 2.3 seconds ± 1.1. These numerical results may not transfer to a population of stroke subjects. Besides, even though we developed our application in collaboration with rehabilitation experts, further studies with actual actions is required to evaluate its application on the target population and the transferability of our results. Finally, while using smaller target sizes did not increase the difficulty, it may be possible that smaller objects than the ones we used could make the game more challenging.

## Conclusion

We presented the results from a user study comparing the effect of first-person and third-person user perspectives, under varying cognitive load, on user performance and upper-extremity movements for a set of reaching tasks in a virtual reality theater environment. Also, we analyzed the effect of user perspectives on the degree of engagement using a questionnaire. Considering only user perspective, the first-person perspective required the participants to make more head movements compared to the third-person perspective. For upper-extremity motion, the interaction between user perspective and higher-cognitive load exercises influenced the movements. These head and upper-extremity movements are essential when assessing the range of motion suitable for VR-based rehabilitation therapy. For user performance, the user perspective and the type of exercise influenced the completion time and the scores. Finally, as hypothesized initially, the user perspective did not influence the self-perceived engagement for this set of relatively easy tasks.

## Methods

### Participants

Thirty students (8 women and 22 men), aged 18–32 years (*M*=24.6 years, SD = 3.6 years) from the University of Illinois at Chicago were recruited for the user study (UIC IRB #2016-0332). Inclusion criteria included being over 18 years old, being an enrolled student, and not having significant physical impairments. We asked every participant about their physical conditions (e.g., movement problems in their trunk, upper-body or head) and their ability to perceive objects in 3D. We explained that any issue would limit their experience with the environment and would affect the expected results. The participants self-reported not having any physical condition that could limit their experience with the environment. Also, none of them reported having stereoscopic vision problems (i.e., problems perceiving depth cues).

### Apparatus and materials

#### CAVE2 environment

**Fig. 7:**
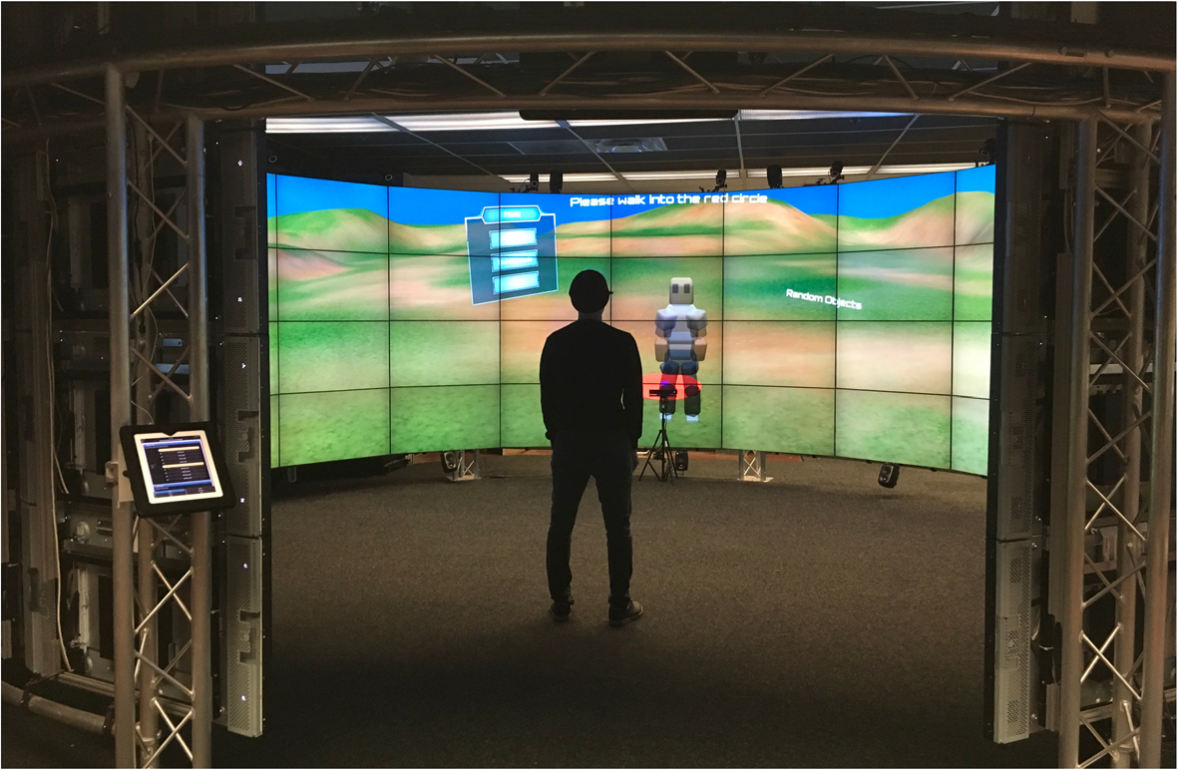
CAVE2 environment. View of the CAVE2 environment, displaying the third-person perspective of a task. 72 high-resolution displays are arranged in eighteen columns in a circular fashion, providing an area of 320 degrees of screen space with a resolution of 36 Megapixels for each user eye

The CAVE2 environment [10] is a large-scale VR environment composed of 72 high-resolution displays arranged in eighteen columns in a circular fashion (Fig 7). The arrangement provides an area of 320 degrees of screens with a resolution of 36 Megapixels per eye. Fourteen Vicon Bonita infrared cameras track the position of retroreflective markers in tracking glasses and controllers in an area of 34 square meters. Twenty speakers and two subwoofers mounted at the top and bottom of the columns provide stereo 3D sound.

A Kinect v2 *Ⓡ* sensor is located in front of the central display (73 cm from the ground) to track the subject upper-extremity joints; the Vicon cameras only track the head position. In our study, to provide quality tracking points for the Kinect sensor, each subject was required to stand in the center of the environment approximately 3.3 meters away from the screens.

**Fig. 8:**
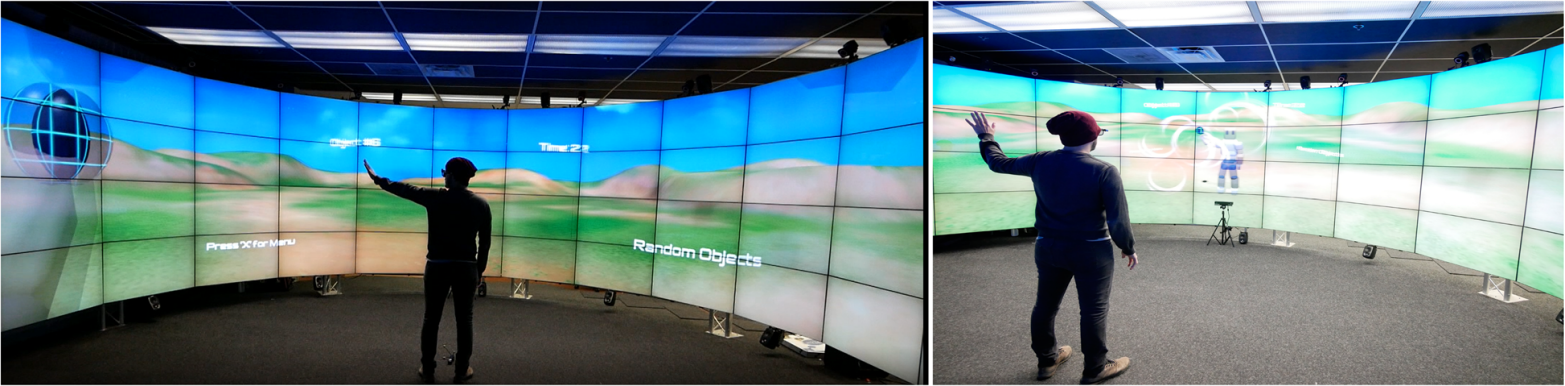
Rehabilitation application. A nonintrusive immersive application of reaching out and touching stationary objects in space, designed to allow comparison of first-person (left) and third-person (right) perspectives in VR-based rehabilitation

**Fig. 9:**
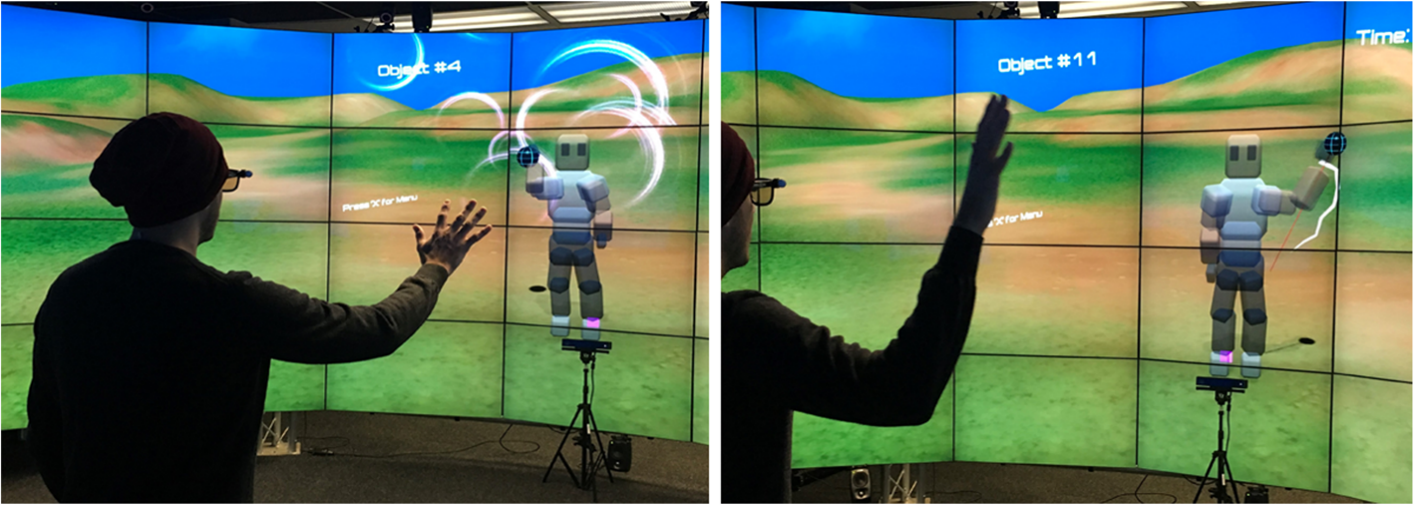
Exercise modes. Exercises in flipped mode (left) and trail mode (right). The flipped mode (left) requires users to use the opposite side to manipulate the avatar correct side (i.e., right physical hand activates left virtual hand, and vice-versa), which requires a higher cognitive load. The trail mode (right) shows a trajectory path to help guide and document the user motion

We designed and implemented a prototype of a VR-based rehabilitation game focused on upper-extremity tasks using Unity3D 5.2.3. The main exercise consists of reaching out with an upper-extremity in order to touch virtual spheres that appear at positions drawn from a uniform distribution, 70 cm away from the player’s chest. The virtual spheres appear one at the time. Experimenters can test different gaming conditions by choosing between first-person and third-person perspectives (Fig. 8). Experimenters can also select the number of targets to reach to. In order to increase or decrease the difficulty of the exercise, experimenters can also vary the target size. To further investigate the role of cognitive function on the user performance under the two perspectives, the prototype implements three exercise types (Fig. 9): 1) easy: normal view; 2) difficult: flipped (the left limb actions moved the right limb, and vice versa); and 3) very easy: trail—a mode that displays a trajectory path and a trace of the hand’s movements to help guide and document the upper-extremity motion.

The CAVE2 environment has, like most second-generation, modern immersive theaters, no ceiling or floor projection. This is due partly to display-technology constraints (while providing remarkably higher resolution and brighter environments than older systems with projected light, displays cannot provide passive/active stereo for multiple users at ceiling or floor locations), and partly due to collaboration requirements (for collaborative use, analysts wish to roll in desks and chairs to create a comfortable-enough environment; in this way, in addition to the environment immersive capabilities, they can continue to use their laptops and web-based technologies as they do in the office). Because of this design, the field of view in the first-person perspective is restricted; subjects only see their hands for fully extended arm elevations between 90 to 120 degrees approximately. Additionally, in the first person perspective the associated trail in trail mode is naturally only visible within the field of view. Object placement is constrained to be always inside the screen-visible space. We use the same space constraint for the third-person perspective, in order to compare both views under the same conditions. Moreover, we used a transparent material for the avatar body, to avoid potential occlusion issues for objects appearing in front of the avatar in the third-person perspective. Before running the experiment, we verified that the distance of an object was perceived similarly on both user perspectives by placing a styrofoam sphere at different locations and a virtual sphere at the same location in the virtual scene.

#### Data

Movement logs from each exercise were recorded in JSON format by the application. The data was preprocessed using Python scripts and further analyzed using R for the statistical tests and G*Power for the power analysis. In addition to the movement logs, we recorded each user study session using a wide lens camera.

### Study design

To determine whether the user perspective and the training settings have an effect on the user performance and body movements in the reaching-objects rehabilitation task, we designed twelve exercises. These activities combined the two user perspectives (first-person and third-person), the three exercise types (normal, flipped [i.e., right physical upper-extremity activates left virtual upper-extremity, and vice-versa], and trail), and two target sizes (spheres of 10 and 15 cm in diameter). Each exercise presented 20 targets, one at a time, and gave up to eight seconds to attempt to touch the target before it disappears. At the beginning of each user study session, each subject performed two rounds of exercises that combined the different settings. We updated the CAVE2 parameters to adjust the virtual scene to the subject’s interpupillary distance. Next, we randomly assigned the starting perspective. However, the six exercises within each user perspective had the same fixed order. The subject first worked with bigger targets in normal mode, then changed to the flipped mode, and finished with the trail mode. Finally, each subject repeated the exercises, this time using the smaller targets. The smaller targets were used to increase the difficulty of the game. Arm reaching was selected by our collaborator at the Shirley Ryan AbilityLab in Chicago, IL, as “the simplest full body task appropriate for a stroke survivor”, and thus appropriate for this study. The number and size of targets, the exercise timing, and the exercise frequency were furthermore selected empirically in order to pose a sufficient challenge to a healthy player. Nevertheless, the number and size of targets, the timing, and the frequency could be adjusted per subject.

We derived two user performance metrics: the total time required to complete the 20 attempts (completion time) and the score per exercise represented as the number of objects caught. In addition, to obtain body movement metrics, we recorded the subject’s joint positions. Body movement metrics included the total number of meters traveled by the hands and head displacements.

Finally, to determine whether the user perspective had an effect on the perceived level of engagement of the CAVE2 environment for the given task, we used the IEQ questionnaire [4]. As recommended by Jennett et al. [4], we grouped the IEQ questions in sub-domains: basic attention, temporal dissociation, challenge, emotional involvement, and enjoyment. The sum of thirty questions on a Likert scale, from one to five, determines the engagement score. At the end of each study session, subjects filled out one survey per user perspective, and were asked to provide any additional feedback to the experimenter.

### Analysis of data

To analyze the effects on user performance and body movements, we performed a multi-factor within-subjects analysis of variance. We checked the normality assumption for the within-subjects test using the Lilliefors (Kolmogorov-Smirnov) test [22]. Data distributions that passed the tests were analyzed with a parametric ANOVA test. As required, we used the Aligned-Rank test [23] for data distributions that failed the tests. As required for repeated-measures ANOVA tests, in the parametric approach we performed an additional sphericity verification using the Mauchly’s test [24], which guarantees that the variances between all within-subject condition pairs are equal. Results for distributions that violate the sphericity assumption were adjusted using the Greenhouse-Geisser correction [25]. We used *P*<0.05 to determine significant differences. Furthermore, we performed post hoc analysis using pairwise comparisons with the Holm-Bonferroni adjustment [26] for parametric approaches. For nonparametric methods, we used the differences of differences cross-factor contrast test [23] to analyze interactions and pairwise comparisons for independent variables.

For the analysis of the level of engagement, we performed a one-way ANOVA test for data that passed the normality and homogeneity of variance assumptions, and a Kruskal-Wallis test [27] for data that violated the assumptions. As in the previous design, we also used *P*<0.05 to determine significant differences.

## Data Availability

Data are available on request.

## Abbreviations

CAVE: Cave automatic virtual environment
VR: Virtual reality
